# Development and Optimization of a Non-Tissue Culture Hairy Root Transformation System in *Camellia oleifera* Mediated by *Agrobacterium rhizogenes*

**DOI:** 10.3390/plants15182794

**Published:** 2026-09-11

**Authors:** Xiaoge Gao, Peishuo Jiang, Ying Zhang, Jichao Wang, Ziqi Fang, Yaqiong Zou, Yizhou Li, Rui Wang, Qirong Guo

**Affiliations:** 1 College of Forestry and Grassland and College of Soil and Water Conservation, Nanjing Forestry University, Nanjing 210037, China; gaoxiaoge@njfu.edu.cn (X.G.); 1543244325@njfu.edu.cn (P.J.); 2410110620@njfu.edu.cn (J.W.); fangziqi@njfu.edu.cn (Z.F.); zyq123@njfu.edu.cn (Y.Z.); liyizhou@njfu.edu.cn (Y.L.); 2 Co-Innovation Center for Sustainable Forestry in Southern China, Nanjing Forestry University, Nanjing 210037, China; 3 Research Institute of Oil Tea Camellia, Hunan Academy of Forestry, Changsha 410004, China; zhangying@hnlky.cn; 4 Yuelushan Laboratory, Changsha 410004, China

**Keywords:** *Camellia oleifera*, tissue culture-independent, hairy roots, RUBY reporter gene, vacuum infiltration

## Abstract

*Camellia oleifera* is an important woody oil crop species endemic to China. However, gene functional analysis and molecular breeding in oiltea are greatly constrained by the low efficiency of conventional *Agrobacterium*-mediated transformation systems. In this study, a rapid and efficient *Agrobacterium rhizogenes*-mediated hairy root transformation system was established using the elite cultivar ‘Xianglin 210’. A vector carrying the RUBY visual reporter gene was introduced into leaf and hypocotyl explants, and the transformation efficiencies of three *Agrobacterium* strains (GV3101, MSU440, and K599) were systematically evaluated. Key transformation parameters, including bacterial suspension density and vacuum infiltration duration, were further optimized. The results demonstrated that leaf explants exhibited significantly higher transformation efficiency than hypocotyl explants. Among the tested strains, K599 showed the highest hairy root induction efficiency. The optimal transformation conditions were obtained using a bacterial suspension at OD_600_ = 0.8 combined with vacuum infiltration for 40 min. Under these conditions, the hairy root induction rate reached 69.00%, and the proportion of RUBY-positive hairy roots reached 58.00%. Notably, the entire transformation process was achieved without callus induction or plant regeneration, and stable transgenic hairy roots were obtained within 60 days. In conclusion, this study demonstrates a rapid and tissue culture-independent hairy root transformation protocol for *C. oleifera*. While further functional studies are needed, this platform provides a practical tool with strong potential for future candidate gene validation and functional genomics in oil tea crops.

## 1. Introduction

*Camellia oleifera* is an economically important woody oil crop species endemic to China. Its seeds are rich in high-quality edible oil, with unsaturated fatty acids accounting for more than 90% of the total fatty acid content, conferring substantial economic, nutritional, and ecological value. Consequently, *C. oleifera* oil is often referred to as “Oriental olive oil” [1,2]. With the rapid advancement of whole-genome sequencing and multi-omics studies, including transcriptomics and metabolomics, a large number of candidate genes associated with oil biosynthesis, stress responses, nutrient acquisition, and root development have been identified in *C. oleifera* [3,4,5,6,7]. The increasing availability of genome-wide resources, transcriptomic datasets, and other omics-based approaches has substantially expanded the discovery of candidate genes and genetic loci underlying agronomically important traits, providing an important foundation for molecular breeding in *C. oleifera*. However, the identification of candidate genes alone is insufficient to establish their biological functions or breeding value. Functional validation of these candidates remains an essential step for translating genomic discoveries into molecular breeding strategies [7,8].

However, due to the lack of an efficient genetic transformation platform, functional characterization of these candidate genes still relies largely on heterologous systems such as *Arabidopsis thaliana*, which has severely hindered functional genomics research and molecular breeding efforts in *C. oleifera* [9,10]. In particular, efficient transformation systems are critical for validating candidate genes identified through genome-wide analyses, transcriptomics, association studies, and comparative genomics, because they enable direct assessment of gene function in the target species and genetic background. The establishment of a rapid and reliable transformation platform would therefore provide an important experimental link between candidate-gene discovery and functional validation, thereby accelerating the application of genomics to molecular breeding in *C. oleifera*.

*Agrobacterium*-mediated transformation remains one of the most widely used approaches for plant functional genomics and genetic improvement. Conventional *A. tumefaciens*-mediated stable transformation generally depends on tissue culture procedures, including callus induction, shoot regeneration, and selection of transformed plants [11,12]. However, the establishment of stable transformation systems in perennial woody species such as *C. oleifera* is particularly challenging because of severe explant browning, low regeneration efficiency, and strong genotype dependence [13]. In *C. oleifera*, considerable variation in somatic embryogenesis and organ regeneration capacity has been reported among different cultivars, resulting in low transformation efficiency, long experimental cycles, and poor reproducibility of conventional tissue culture-based transformation methods [14,15]. Several researchers have employed virus-induced gene silencing (VIGS) to silence *CdCRY1* and *CdLAC15* in *C. drupifera*, and verified that these genes modulate anthocyanin accumulation in fruits [16]. Nevertheless, this assay only achieved transient functional validation in fruit peels, and stable transgenic lines were not obtained. These limitations have significantly restricted the application of molecular approaches for gene functional analysis and genetic improvement in this species.

In recent years, *A. rhizogenes*-mediated hairy root transformation has emerged as an effective alternative for functional studies in recalcitrant plant species because it bypasses the need for plant regeneration and substantially shortens the transformation cycle [17]. Following integration of *Ri* plasmid T-DNA into the host genome, transgenic hairy roots can be rapidly induced and maintained with high genetic stability, providing a convenient platform for gene functional analysis [18,19]. This approach has been successfully applied in several woody species, including *Passiflora edulis*, *Actinidia chinensis*, and *Pyrus ussuriensis*, and has been widely utilized to investigate root development, stress-response mechanisms, and secondary metabolism [20,21,22]. Within the Theaceae family, such a system has been reported in *C. sinensis*. Using *A. rhizogenes* strain ATCC15834 carrying a GFP reporter gene, transgenic hairy roots can be obtained within 35–50 days, with an overall experimental cycle of only two months and no requirement for antibiotic selection steps. This system achieved the first CRISPR/Cas9 genome-editing in tea plant. Through overexpression and editing of *CsMYB73*, this transcription factor was confirmed to negatively regulate theanine biosynthesis; the theanine content was significantly reduced in transgenic roots overexpressing *CsMYB73* [23]. Nevertheless, despite these successful applications within Theaceae, no efficient hairy root transformation system has been reported for *C. oleifera*.

In addition to the choice of bacterial strain and explant type, infection methods and screening strategies are critical factors affecting transformation efficiency. Vacuum infiltration has been demonstrated to enhance *Agrobacterium* penetration into plant tissues by reducing internal air pressure, thereby increasing bacterial contact with target cells and improving T-DNA transfer efficiency, particularly in woody plants with dense tissue structures [23,24]. Furthermore, the recently developed RUBY visual reporter system enables the accumulation of betalain pigments in transgenic tissues, resulting in a visible red-purple phenotype that can be directly observed without fluorescence microscopy or histochemical staining [25]. Compared with conventional reporter systems such as GUS and GFP, RUBY offers several advantages, including low cost, simple operation, and rapid non-destructive screening, making it a promising tool for the establishment of efficient transformation systems [26,27].

Given the absence of an efficient, tissue culture-independent transformation platform in *C. oleifera*, the present study aimed to establish a rapid and highly reproducible *A. rhizogenes*-mediated hairy root transformation system using the elite cultivar ‘Xianglin 210’. Leaf and hypocotyl explants were employed to evaluate transformation performance. By comparing the transformation efficiencies of different *A. rhizogenes* strains, optimizing key parameters such as bacterial density and vacuum infiltration duration, and integrating the RUBY visual reporter system for rapid screening, we sought to develop a simple, efficient, and reproducible transformation method for *C. oleifera*. Beyond protocol development, this platform is intended to provide a rapid functional-validation system for candidate genes identified through genomic and multi-omics approaches, particularly those involved in root development, nutrient acquisition, stress responses, and other agriculturally important traits. By enabling efficient gene overexpression and functional analysis directly in *C. oleifera* tissues, the established system may help bridge the gap between genomic candidate-gene discovery and functional genomics, and ultimately facilitate the utilization of genomic resources in molecular breeding of this economically important woody oil crop.

## 2. Results

### 2.1. Establishment of a Tissue Culture-Independent Hairy Root Transformation System in C. oleifera

**Figure 1:**
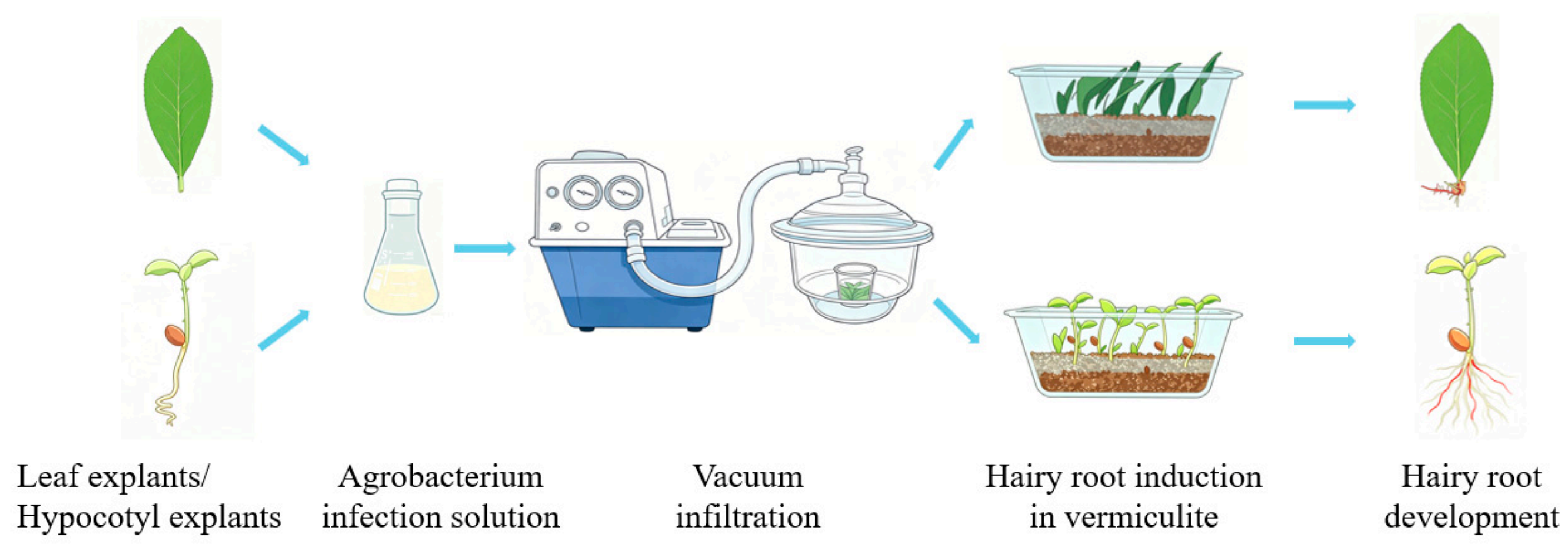
Schematic diagram of the *Agrobacterium*-mediated transformation system for oiltea hairy roots.

To establish a tissue culture-independent genetic transformation system for *C. oleifera*, young seedlings of the cultivar ‘Xianglin 210’ were used as experimental materials. Leaf and hypocotyl explants were subjected to *Agrobacterium*-mediated transformation followed by cultivation in vermiculite substrate for hairy root induction (Figure 1). The transformation procedure consisted of explant preparation, bacterial suspension preparation, vacuum infiltration, co-cultivation, hairy root induction, and screening of transgenic roots.

**Figure 2:**
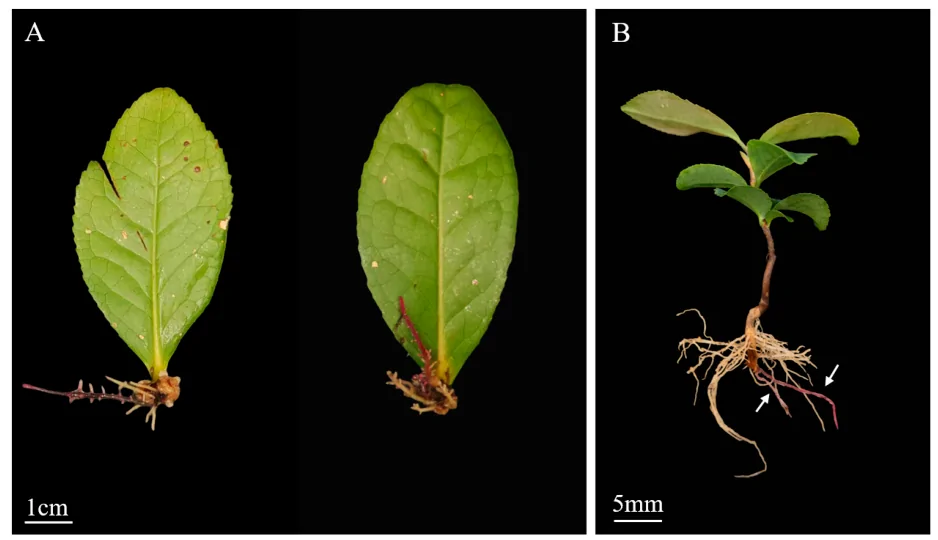
Phenotypes of hairy roots from different oiltea explants following *Agrobacterium*-mediated transformation. (**A**): RUBY-positive hairy roots induced from leaf explants; (**B**): RUBY-positive hairy roots induced from hypocotyl explants. The transgenic hairy roots accumulate betacyanin pigments and exhibit a distinct red color, whereas the non-transgenic roots remain white.

At 60 days after infection, hairy roots were successfully induced from wound sites of both leaf and hypocotyl explants (Figure 2). Hairy roots carrying the RUBY reporter gene accumulated betalain pigments and exhibited a distinct red coloration, whereas non-transgenic roots remained creamy white. This visible phenotype enabled direct identification of transgenic roots without fluorescence detection or histochemical staining. Notably, the entire procedure was completed without callus induction or plant regeneration, and transgenic hairy roots suitable for subsequent molecular analyses were obtained within 60 days.

To evaluate the effect of explant type on transformation efficiency, the number of rooting explants, hairy root induction rate, and RUBY-positive transformation rate were quantified under identical transformation conditions (Table 1). Leaf explants produced an average of 11.00 rooting explants, which was significantly higher than that observed in hypocotyl explants. The hairy root induction rate of leaf explants reached 22%, representing a 3.1-fold increase compared with hypocotyl explants, and the difference was statistically significant (*p* < 0.05). The RUBY-positive transformation rate was 25% in leaf explants and 11% in hypocotyl explants; however, no significant difference was detected between the two explant types (*p* > 0.05).

Morphological observation further revealed differences in hairy root growth between explant types (Figure 2). Hairy roots induced from leaf explants exhibited vigorous growth and strong RUBY pigmentation, whereas transformed roots derived from hypocotyl explants were relatively thin and displayed visible RUBY coloration mainly at the root tips. Overall, leaf explants exhibited higher hairy root induction efficiency and generated more robust transgenic hairy roots than hypocotyl explants under the tested conditions.

### 2.2. Effects of Different Agrobacterium Strains on Hairy Root Induction and Transformation Efficiency

To evaluate the effects of different *Agrobacterium* strains on hairy root induction and transformation efficiency, the p3302-RUBY construct was introduced into three strains, GV3101, MSU440, and K599. Leaf explants were infected under identical conditions, and hairy root induction and transformation efficiencies were subsequently assessed (Table 2).

Significant differences in transformation performance were observed among the three strains. Hairy roots were induced by all strains; however, GV3101 exhibited a markedly lower induction capacity than the other two strains. The average number of rooting explants obtained with GV3101 was 1.33, with a hairy root induction rate of only 3%. In contrast, MSU440 and K599 showed substantially higher induction efficiencies, producing an average of 17.67 and 20.67 rooting explants, respectively. The corresponding hairy root induction rates reached 35% and 41%, both significantly higher than those obtained with GV3101 (*p* < 0.05).

Transformation efficiency was further evaluated using RUBY-positive hairy roots as an indicator. No RUBY-positive roots were detected in GV3101-treated explants. In contrast, K599 achieved the highest RUBY-positive transformation rate (24%), followed by MSU440 (14%). Although K599 consistently exhibited higher values than MSU440 in terms of hairy root induction rate, average number of rooting explants, and RUBY-positive transformation rate, the differences between the two strains were not statistically significant (*p* > 0.05).

Taken together, K599 exhibited the highest overall performance for hairy root induction and transgenic root production among the tested strains and was therefore selected for subsequent optimization experiments.

### 2.3. Optimization of A. rhizogenes K599 Infection Conditions

**Figure 3:**
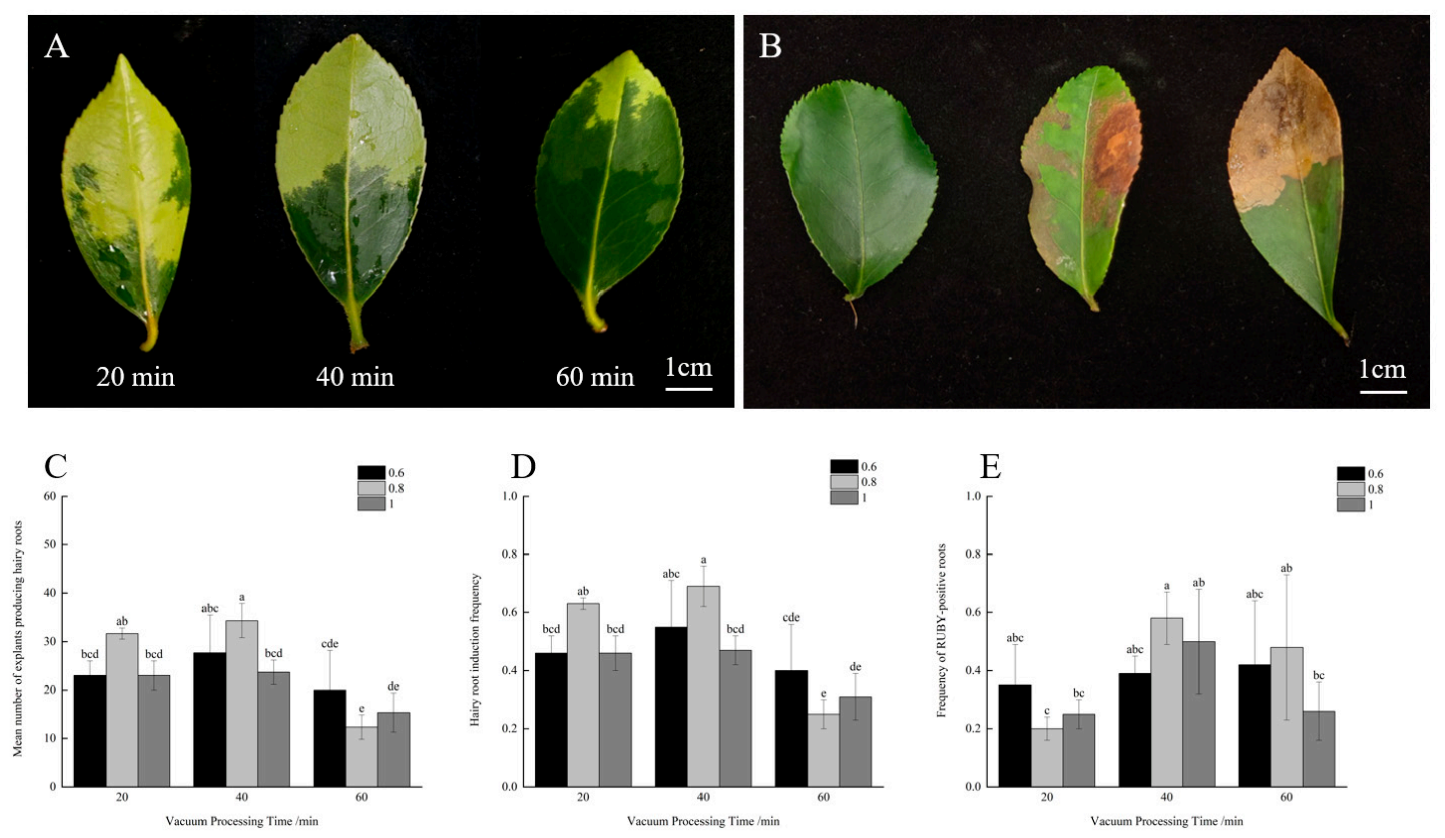
Phenotypic performance and transformation efficiencies of *C. oleifera* leaf explants under varied *Agrobacterium* infection conditions. (**A**) Representative leaf explant phenotypes under OD_600_ = 0.8 with 20 min, 40 min, and 60 min vacuum infiltration, respectively. (**B**) Representative necrotic leaf phenotypes caused by excessive infection stress. (**C**) Mean number of explants producing hairy roots per biological replicate. (**D**) Model-estimated marginal mean of hairy-root induction rate. (**E**) Model-estimated marginal mean of RUBY-positive transformation rate. For panels (**C**–**E**), error bars represent standard error (SE) from binomial generalized linear models. Different lowercase letters indicate significant differences among all nine treatment combinations by Tukey-adjusted pairwise comparisons (*p* < 0.05). Treatments sharing at least one common letter are not statistically significantly different.

To determine the optimal infection conditions for hairy root transformation in *C. oleifera*, the strain K599 and leaf explants were used for further optimization. Three bacterial suspension densities (OD_600_ = 0.6, 0.8, and 1.0) and three vacuum infiltration durations (20, 40, and 60 min) were evaluated in a factorial experiment (Table 3, Figure 3). Obvious phenotypic differences were observed in leaf explants after different vacuum infiltration durations (Figure 3A).

Binomial generalized linear model (GLM) analysis revealed that both bacterial suspension density and vacuum infiltration duration significantly affected hairy root induction efficiency, with a significant interaction between these two factors, indicating that the effect of one factor depended on the level of the other. At 40 min infiltration, the OD_600_ = 0.8 treatment yielded significantly higher hairy-root induction rates relative to OD_600_ = 0.6 and 1.0, whereas this difference was only marginally significant at 20 min. Among all treatment combinations, OD_600_ = 0.8 combined with 40 min of vacuum infiltration produced the highest induction efficiency, yielding an average of 34.33 rooting explants and a hairy root induction rate of 69%, both significantly higher than those observed in the other treatments (Figure 3C,D).

Consistent with the significant interaction, the effect of vacuum infiltration duration varied with bacterial density. At the same bacterial concentration, hairy root induction generally increased from 20 to 40 min and subsequently decreased at 60 min. For example, under OD_600_ = 0.8, the hairy root induction rate increased from 63% at 20 min to 69% at 40 min, before declining to 25% at 60 min. A similar trend was observed for the average number of rooting explants, which increased from 31.67 to 34.33 and then decreased to 12.33 as the infiltration duration increased from 20 to 60 min (Figure 3C,D).

Analysis of RUBY-positive transformation rates further supported these results. Likewise, a significant interaction between bacterial density and infiltration duration was detected for RUBY-positive transformation rate, even though the main effect of OD_600_ was non-significant. The highest RUBY-positive transformation rate (58%) was achieved under the combination of OD_600_ = 0.8 and 40 min of vacuum infiltration. In contrast, although the treatment with OD_600_ = 0.8 and 20 min of vacuum infiltration produced a relatively high hairy root induction rate (63%), its RUBY-positive transformation rate was only 20% (Figure 3E). No other treatment combination achieved comparable levels of both hairy root induction and RUBY-positive transformation efficiency. In addition, excessive bacterial concentration and overlong vacuum treatment caused severe leaf browning and necrosis (Figure 3B), which greatly reduced the survival rate of explants.

Overall, the combination of a bacterial suspension density of OD_600_ = 0.8 and 40 min of vacuum infiltration resulted in the highest hairy root induction and transformation efficiencies and was therefore identified as the optimal condition for *A. rhizogenes*-mediated hairy root transformation in *C. oleifera*.

### 2.4. Molecular Verification of Transgenic Hairy Roots

**Figure 4:**
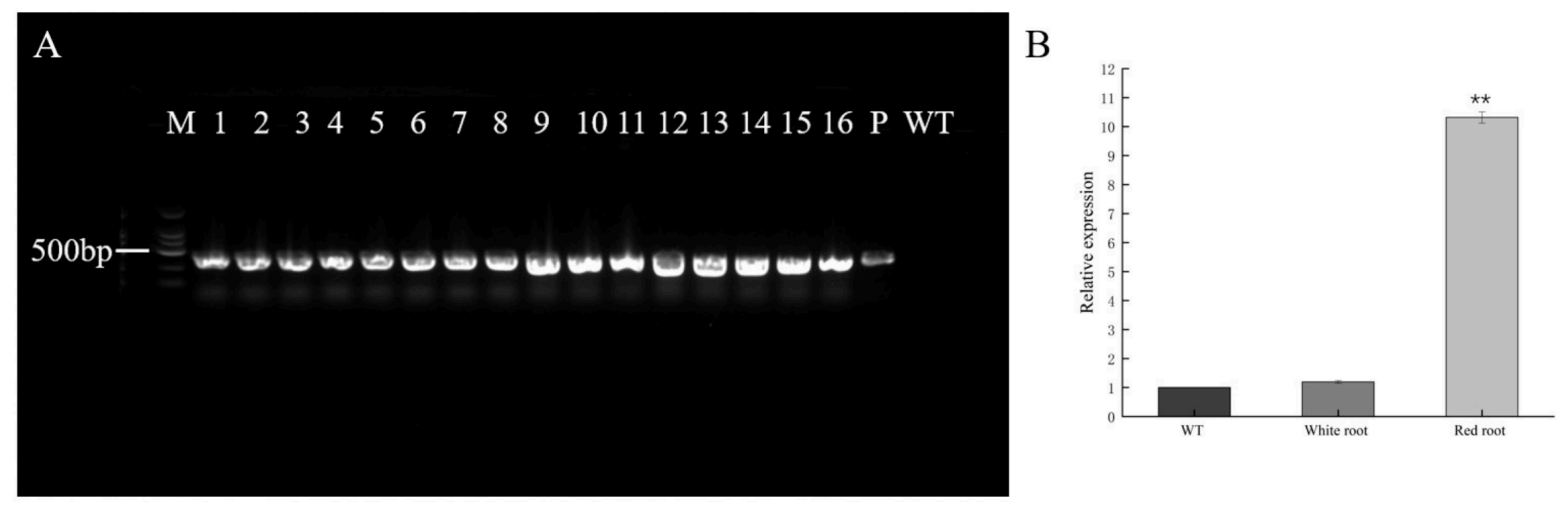
(**A**) PCR-based molecular validation of RUBY-positive transgenic hairy roots in oiltea. M: DNA marker; P: plasmid positive control; WT: wild-type root control; 1–16: independent transgenic hairy root lines. The expected amplicon size was 368 bp. (**B**) Relative expression of RUBY in WT, white and red hairy roots. Asterisks indicate significant differences (** *p* ≤ 0.01).

To verify the transgenic nature of RUBY-positive hairy roots, sixteen independent RUBY-expressing hairy root lines obtained under the optimal transformation conditions were randomly selected for molecular analysis. Genomic DNA was extracted from each line and subjected to PCR amplification using RUBY-specific primers (Figure 4A).

As expected, a specific DNA fragment of 368 bp corresponding to the RUBY gene was successfully amplified from the plasmid positive control and all sixteen RUBY-positive hairy root lines (lanes 1–16). In contrast, no amplification product was detected in the wild-type root sample (WT). Furthermore, no non-specific amplification or bands indicative of residual *Agrobacterium* contamination were observed. These results confirmed the stable integration of the RUBY transgene into the genome of the transformed hairy roots. The PCR results were fully consistent with the visible RUBY pigmentation phenotype, indicating that the RUBY reporter system provides an effective and reliable approach for the identification of transgenic hairy roots in *C. oleifera*.

After molecular identification by genomic PCR, red-pigmented hairy root lines were selected for qRT-PCR analysis to characterize the transcript level of RUBY. Non-pigmented white hairy roots derived from the same *Agrobacterium*-infected hypocotyl explants, together with uninfected WT roots, were used as controls. The qRT-PCR results indicated that the transcript abundance of RUBY was significantly up-regulated in red-pigmented hairy roots. No significant difference was observed between the signal detected in white hairy roots and the background level of wild-type roots. These results confirm that the red phenotype is driven by high-level RUBY transcription, and positive transgenic hairy roots are verified at the transcriptional level (Figure 4B).

## 3. Discussion

### 3.1. Tissue Culture-Independent Transformation Provides a Novel Approach for Functional Gene Studies in C. oleifera

With the rapid advancement of genome sequencing and multi-omics studies in *C. oleifera*, an increasing number of candidate genes have been identified and require functional validation through genetic transformation. However, the development of molecular biology research in *C. oleifera* has been severely constrained by the limitations of conventional tissue culture-based transformation systems, including low regeneration efficiency, prolonged experimental cycles, and strong genotype dependence. In the present study, a tissue culture-independent hairy root transformation system was established that bypasses the callus induction and plant regeneration processes. Stable transgenic hairy roots were obtained directly from conventionally grown seedlings, substantially reducing the time and technical complexity required for transformation.

In recent years, *A. rhizogenes*-mediated hairy root transformation has been widely applied in woody plant species; however, its application in *C. oleifera* remains limited [23,28,29]. The present study demonstrates that this approach is also suitable for *C. oleifera* and exhibits satisfactory reproducibility and stability. The established system provides a valuable platform for the rapid functional characterization of genes involved in root development, nutrient uptake, stress responses, and secondary metabolism.

It should be noted that the transformed materials generated in this study are composite plants, in which only the root system is transgenic while the aerial tissues remain non-transformed. Consequently, this system is particularly suitable for investigating root development, nutrient acquisition, rhizosphere interactions, and stress-response mechanisms. However, it may be less suitable for studies requiring evaluation of whole-plant phenotypes. Future efforts could integrate grafting systems, hairy root regeneration approaches, or genome-editing technologies to expand the application of this platform in molecular breeding programs for *C. oleifera*.

### 3.2. Explant Type Is a Critical Determinant of Hairy Root Transformation Efficiency in C. oleifera

The choice of explant is one of the most important factors affecting *A. rhizogenes*-mediated transformation efficiency. In this study, leaf explants exhibited higher hairy root induction rates and RUBY-positive transformation frequencies than hypocotyl explants. Studies in *Prunus persica*, *Idesia polycarpa*, and *Citrullus colocynthis* have illustrated that the most suitable explant type varies across plant species, and tissue background plays a crucial role in determining susceptibility to *A. rhizogenes* infection [30,31,32].

The superior performance of leaf explants may be attributed to their anatomical and physiological characteristics. Leaf tissues generally contain a higher proportion of parenchyma cells with greater mitotic activity, facilitating bacterial attachment and T-DNA transfer. In addition, the petiole base possesses relatively high organogenic competence and may be more responsive to root primordium induction mediated by *rol* genes. By contrast, hypocotyl tissues often exhibit a higher degree of lignification and thicker cell walls, which may impede bacterial penetration and reduce transformation efficiency [33]. These findings indicate that leaf explants are more suitable recipient materials for tissue culture-independent transformation in *C. oleifera* and may serve as the preferred explant type for future transformation studies.

### 3.3. K599 Exhibits Superior Infectivity and Transformation Performance in C. oleifera

Transformation efficiency is strongly influenced by the bacterial strain employed. In the present study, three *Agrobacterium* strains, GV3101, MSU440, and K599, were evaluated, and K599 consistently produced the highest hairy root induction rate and RUBY-positive transformation frequency. Similar results have been reported in other woody species, including *Syringa pinnatifolia*, *Ziziphus jujuba*, and *Eriobotrya japonica* [28,34,35], suggesting that K599 possesses broad applicability for woody plant transformation.

Previous studies have demonstrated that differences in Ri plasmid composition and virulence gene expression among *A. rhizogenes* strains can directly influence T-DNA transfer efficiency [36]. K599, which originates from the A4 Ri plasmid lineage, is characterized by strong root-inducing capability. Its high infectivity and favorable host compatibility likely contribute to the superior transformation efficiency observed in this study [37,38]. Nevertheless, host–bacterium interactions are often genotype-dependent. In related studies on *C. sinensis*, the *A. rhizogenes* strain ATCC15834 outperformed K599, achieving a transformation efficiency of 11.39% using leaf explants via vacuum infiltration [23]. In other woody plant species, the transformation performance varies considerably depending on explant types and *Agrobacterium* strains (Table 4). Therefore, although K599 proved highly effective in the cultivar ‘Xianglin 210’, further studies are required to determine whether similar transformation efficiencies can be achieved in other *C. oleifera* cultivars.

### 3.4. Vacuum Infiltration Significantly Enhances Agrobacterium Infection Efficiency

Increasing effective contact between *Agrobacterium* cells and plant tissues is a key strategy for improving transformation efficiency. Through optimization of bacterial suspension density and vacuum infiltration duration, the present study established suitable infection conditions for hairy root transformation in *C. oleifera*. The results demonstrated that an appropriate bacterial concentration can balance bacterial viability and explant survival, while pre-induction of bacterial suspensions following resuspension may enhance vir gene activation and thereby improve transformation competence. Vacuum infiltration further increased transformation efficiency by facilitating penetration of the bacterial suspension into internal leaf tissues and enhancing T-DNA delivery. Similar effects have been reported in sunflower and soybean transformation systems [45,46]. However, prolonged vacuum treatment reduced transformation efficiency, likely due to excessive tissue dehydration and cellular damage. Likewise, excessively high bacterial densities may lead to bacterial overgrowth, triggering hypersensitive responses and tissue necrosis, ultimately reducing transformation success (Figure 3B) [47,48].

These findings highlight the importance of optimizing infection parameters for the development of efficient and reproducible transformation systems in woody plants. Although a relatively high transformation efficiency was achieved in this study, additional improvements may still be possible. Future investigations could evaluate sonication-assisted *Agrobacterium* transformation, alternative surfactants and their concentrations, vacuum pressure gradients, acetosyringone concentrations, and explants collected at different developmental stages.

### 3.5. The RUBY Visual Reporter System Improves Screening Efficiency

Reporter genes are important tools for evaluating plant transformation efficiency. Conventional reporters, such as GUS, GFP, and luciferase (LUC), have been widely used, but each has practical limitations. GUS detection generally requires destructive histochemical staining with substrates such as X-Gluc, whereas GFP requires fluorescence excitation and detection equipment and may be affected by tissue autofluorescence. LUC is highly sensitive and quantitative but requires exogenous luciferin and specialized luminescence-detection equipment [49].

RUBY provides a convenient visual alternative by converting tyrosine into red betalain pigments through a three-enzyme biosynthetic pathway. The resulting red pigmentation can be readily detected by the naked eye without destructive staining, exogenous substrates, or specialized imaging equipment. RUBY has been successfully applied for plant transformation and, more recently, to *A. rhizogenes*-mediated hairy root transformation, including in the *Jojoba*, *S. pinnatifolia* [34,42]. These features make RUBY particularly suitable for rapid and non-destructive screening of transgenic hairy roots in *C. oleifera*.

In the present study, the RUBY visual reporter system enabled direct identification of transgenic tissues through the accumulation of betalain pigments. PCR analysis confirmed the presence of the expected RUBY-specific amplicon in all tested RUBY-positive hairy root lines, indicating a high level of consistency between visible pigmentation and transgene integration. This finding is consistent with the original report describing the RUBY system and demonstrates its applicability for transformation screening in woody plant species [25].

Despite these advantages, several limitations should be considered. Betalain accumulation can be influenced by promoter activity, transgene insertion position, and the availability of metabolic precursors such as tyrosine, potentially resulting in variation in pigmentation intensity among independent transformation events. Consequently, transformants lacking visible red pigmentation (white hairy roots) show RUBY expression levels nearly identical to wild-type (WT) roots, as validated by our qPCR analysis of WT, white, and red hairy roots (Figure 4B). The RUBY transcript abundance was drastically higher in red hairy roots, with significant differences compared with WT and white hairy roots (*p* ≤ 0.01). These findings demonstrate that white hairy roots are non-transgenic, while red pigmentation reliably indicates positive transformation. Nevertheless, additional molecular verification by PCR or qPCR is still recommended to confirm transgenic events. Therefore, RUBY is best regarded as a rapid preliminary screening tool rather than a complete substitute for molecular analyses.

Overall, the integration of the RUBY reporter system with the hairy root transformation platform substantially improved the efficiency of transgenic material identification and provides a valuable foundation for future gene-editing and functional genomics studies in *C. oleifera*.

### 3.6. Limitations and Future Perspectives

Despite its high efficiency and reproducibility, several limitations of this tissue culture-independent hairy root transformation system should be acknowledged. Optimization was primarily performed using the elite cultivar ‘Xianglin 210’. Preliminary experiments indicated that the protocol could also be applied to other cultivated and wild *Camellia* species, including *C. crapnelliana*, *C. lanceoleosa*, and *C. hainanica*. However, systematic validation across a broader range of genotypes and genetic backgrounds is still required, and further optimization may be necessary for specific *Camellia* germplasms. Future studies should therefore focus on expanding the applicability of this platform and evaluating its compatibility with downstream approaches, including gene overexpression, RNA interference, and CRISPR/Cas9-mediated gene editing.

Beyond its application in hairy root induction, this rapid transformation system provides a potential platform for functional characterization of genes in *C. oleifera*. Compared with conventional stable transformation, which can be time-consuming in woody plants, the approximately 60-day hairy root protocol may substantially reduce the time required for preliminary functional evaluation. The system could facilitate rapid screening of candidate genes identified through genomic and multi-omics analyses, as well as the characterization of genes involved in root development, nutrient uptake, and abiotic stress responses. In addition, coupling candidate promoters or genes with suitable reporter systems may enable the evaluation of promoter activity and gene function in transformed roots. The platform may also provide a useful experimental system for investigating metabolic processes in roots, including secondary metabolite biosynthesis and lipid-related pathways. Further integration with genome editing and other functional genomics approaches could expand its utility for gene function validation and accelerate the translation of genomic discoveries into molecular breeding applications in *C. oleifera* and other woody *Camellia* species.

## 4. Materials and Methods

### 4.1. Plant Materials and Growth Conditions

Seeds of the elite C. oleifera cultivar ‘Xianglin 210’ were germinated and sown in seedling substrate. Seedlings were grown in a controlled-environment growth chamber at Nanjing Forestry University under a 16 h light/8 h dark photoperiod, a temperature of 24 ± 2 °C, relative humidity of 70–85%, and a light intensity of approximately 2500 lx. The substrate was maintained under adequate moisture throughout the cultivation period.

When two fully expanded true leaves had developed, healthy and uniformly growing seedlings were selected for transformation experiments. Mature leaves with intact petioles and hypocotyl segments were used as explants.

### 4.2. Plant Expression Vector and Agrobacterium Strains

The vector p3302-RUBY (Chongqing BioGround Biotechnology Co., Ltd., Chongqing, China) was used in this study. The RUBY reporter cassette consists of three betalain biosynthetic genes (CYP76AD1, DODA, and a glucosyltransferase), which enable visible red pigmentation in transformed tissues.

Three bacterial strains were employed in this study: the Agrobacterium tumefaciens strain GV3101 and two *Rhizobium rhizogenes* strains (syn. *Agrobacterium rhizogenes*), MSU440 and K599 (Shanghai Weidi Biotechnology Co., Ltd., Shanghai, China). For convenience and consistency with the plant transformation literature, these strains are collectively referred to as *Agrobacterium* strains throughout the manuscript. The p3302-RUBY vector was introduced into each strain by the freeze–thaw method. Positive colonies were verified by colony PCR and stored in TY medium containing 15% (*v*/*v*) glycerol at −80 °C until use.

### 4.3. Preparation of Agrobacterium Suspension

Verified *Agrobacterium* strains were streaked onto TY agar plates supplemented with appropriate antibiotics and incubated at 28 °C for 48 h. Single colonies were inoculated into TY liquid medium containing the same antibiotics and cultured at 28 °C with shaking at 220 rpm until the OD_600_ reached 0.8–1.0. Bacterial cultures were collected by centrifugation at 4000× *g* for 10 min and resuspended in infection buffer to the desired OD_600_ values. The infection buffer contained MES, MgCl_2_, Acetosyringone (AS), and 0.01% (*v*/*v*) Silwet L-77, adjusted to pH 5.6. The bacterial suspension was incubated in the dark at room temperature for 3 h before infection to induce virulence gene expression.

### 4.4. Tissue Culture-Independent Hairy Root Transformation

For leaf transformation, whole leaves with intact petioles were collected from young seedlings. The petioles were fully immersed in the Agrobacterium suspension and immediately subjected to vacuum infiltration. For hypocotyl transformation, the primary roots of young seedlings were excised prior to infection. The basal cut surface and lower hypocotyl region were gently wounded using a sterile scalpel.

Explants were subjected to vacuum infiltration for 20, 40, or 60 min and subsequently blotted dry on filter paper to remove excess bacterial suspension. Treated explants were then embedded in vermiculite and co-cultivated in darkness for 3 d. After co-cultivation, explants were transferred to growth chamber conditions as described above. Hairy root emergence was monitored regularly throughout the cultivation period. Hairy root induction time, induction rate, and root growth characteristics were recorded. Hairy roots approximately 2–3 cm in length were used for visual screening and molecular analysis.

Hairy root induction rate was calculated as follows: Hairy root induction rate (%) = (Number of explants producing hairy roots/Total number of infected explants) × 100. This metric reflects the proportion of all inoculated explants that were capable of generating hairy roots.

### 4.5. Screening and Molecular Verification of RUBY-Positive Hairy Roots

Transgenic hairy roots were preliminarily identified based on RUBY-mediated betalain accumulation. RUBY-positive hairy roots displayed a distinct red-purple phenotype, whereas non-transformed roots remained creamy white. The RUBY-positive transformation rate was calculated as follows:

For molecular verification, genomic DNA was extracted from RUBY-positive hairy roots using the CTAB method. PCR amplification was performed using RUBY-specific primers: RUBY-F (5′-ATGAAGCTTATGGCTTCGAT-3′) and RUBY-R (5′-TCATGGATCCCTAGTTGGTG-3′), generating an expected amplicon of 368 bp.

PCR reactions were performed in a total volume of 20 μL containing 10 μL 2× Taq Mix, 0.8 μL each primer, 1 μL genomic DNA template, and nuclease-free water to volume. Amplification conditions were as follows: initial denaturation at 95 °C for 3 min; 35 cycles of 95 °C for 15 s, 58 °C for 30 s, and 72 °C for 20 s; followed by a final extension at 72 °C for 5 min. PCR products were separated on 1.0% agarose gels and visualized using a gel documentation system.

To characterize the transcript levels of RUBY in transgenic hairy roots, total RNA was isolated from red-pigmented hairy roots and paired non-pigmented white hairy roots derived from the identical hypocotyl explants using the Trizol reagent. Following DNase I treatment to eliminate genomic DNA contamination, RNA was reverse-transcribed into cDNA. Quantitative real-time PCR (qPCR) was performed with the SYBR Green I system (Vazyme, Nanjing, China) for gene expression quantification. The length of qPCR amplicons was restricted to 70–150 bp. Relative gene expression was calculated by the 2^−ΔΔCt^ method using *CoActin* as the internal reference gene. Three biological replicates were applied, and each biological sample was assayed in technical triplicate.

### 4.6. Statistical Analysis

All experiments were conducted using three independent biological replicates, with 50 explants per treatment combination in each replicate. Explants of similar developmental stage and size were selected and randomly allocated to the treatment groups.

For single-factor experiments evaluating variables such as explant type and *Agrobacterium* strain, treatment effects were analyzed using generalized linear models (GLMs) with a binomial distribution and logit link function. For the infection-condition optimization experiment, bacterial suspension density (OD_600_ = 0.6, 0.8, and 1.0) and vacuum infiltration duration (20, 40, and 60 min) were assessed using a two-way factorial binomial GLM. Fixed effects in the model comprised bacterial suspension density, vacuum infiltration duration, and their two-way interaction. Overdispersion was assessed for all fitted binomial models, and no substantial overdispersion was observed. When overall significant effects were detected, post-hoc pairwise comparisons were performed on estimated marginal means with Tukey’s adjustment for multiple comparisons.

Statistical significance was defined as *p* < 0.05. Data are presented as mean ± standard error (SE). Statistical analyses were performed in R software (R-4.5.2), and graphs were generated using Origin 2025 (Origin Lab Corporation, Northampton, MA, USA).

## 5. Conclusions

In summary, a highly reproducible, tissue culture-independent *A. rhizogenes*-mediated hairy root transformation system was developed and systematically optimized using *C. oleifera* seedling explants. Experimental evidence demonstrates that: (1) leaf explants outperform hypocotyl explants in induction rate; (2) strain K599 yields superior transformation efficiency compared to MSU440 and GV3101; and (3) a combination of OD_600_ = 0.8 with 40 min vacuum infiltration represents the optimal condition, achieving a 69.00% hairy root induction rate and 58.00% RUBY-positive transformation rate within 60 days. As potential downstream applications, this optimized protocol lays a technical foundation that may facilitate future candidate gene screening, promoter characterization, and nutrient transport research in *C. oleifera*.

## Figures and Tables

**Table 1:** Effects of different explant types on hairy root induction and transformation efficiency in oiltea.

Explant Type	Mean Number of Hairy Root-Producing Explants	Hairy Root Induction Rate	RUBY-Positive Transformation Rate
leaf explants	11.00 ± 3.60 a	0.22 ± 0.07 a	0.25 ± 0.11 a
hypocotyl explants	3.67 ± 2.08 b	0.07 ± 0.04 b	0.11 ± 0.19 a

Values represent raw observed means ± standard error (SE) across biological replicates. Different lowercase letters within each column indicate significant differences between explant types (*p* < 0.05).

**Table 2:** Effects of different *Agrobacterium* strains on hairy root induction and transformation efficiency in oiltea.

*Agrobacterium* Strain	Mean Number of Hairy Root-Producing Explants	Hairy Root Induction Rate	RUBY-Positive Transformation Rate
GV3101	1.33 ± 1.53 b	0.03 ± 0.03 b	0 a
MSU440	17.67 ± 2.08 a	0.35 ± 0.04 a	0.14 ± 0.10 a
K599	20.67 ± 2.08 a	0.41 ± 0.04 a	0.24 ± 0.07 a

Values represent raw observed means ± standard error (SE) across biological replicates. Different lowercase letters within each column indicate significant differences between explant types (*p* < 0.05).

**Table 3:** Effects of vacuum infiltration duration and OD_600_ on hairy root induction and transformation efficiency in oiltea.

Vacuum Infiltration Duration (min)	Bacterial Density (OD_600_)	Mean Number of Hairy Root-Producing Explants	Hairy Root Induction Rate	RUBY-Positive Transformation Rate
20	0.6	23.00 ± 3.00 bcd	0.46 ± 0.06 bcd	0.35 ± 0.14 abc
	0.8	31.67 ± 1.15 ab	0.63 ± 0.02 ab	0.20 ± 0.04 c
	1	23.00 ± 3.00 bcd	0.46 ± 0.06 bcd	0.25 ± 0.05 bc
40	0.6	27. 67 ± 7.77 abc	0.55 ± 0.16 abc	0.39 ± 0.06 abc
	0.8	34.33 ± 3.51 a	0.69 ± 0.07 a	0.58 ± 0.09 a
	1	23.67 ± 2.52 bcd	0.47 ± 0.05 bcd	0.50 ± 0.18 ab
60	0.6	20.00 ± 8.18 cde	0.40 ± 0.16 cde	0.42 ± 0.22 abc
	0.8	12.33 ± 2.52 e	0.25 ± 0.05 e	0.48 ± 0.25 ab
	1	15.33 ± 4.04 ade	0.31 ± 0.08 de	0.26 ± 0.10 bc

Values represent raw observed means ± standard error (SE) derived from binomial generalized linear models. Different lowercase letters within each column indicate significant differences among all nine combined treatment combinations (combinations of vacuum infiltration duration and bacterial OD_600_), according to Tukey-adjusted pairwise comparisons (*p* < 0.05). Treatments sharing at least one common letter are not statistically significantly different. Vacuum infiltration duration and bacterial suspension density (OD_600_) served as experimental factors.

**Table 4:** Recent advances in research on hair roots of woody plants over the past three years.

Species	Agrobacterium	Marker Gene	Conversion Cycle	Transformation State	Positive Efficiency	References
*Syringa pinnatifolia*	K599	RUBY	30	seedling without root–transgenic root	28.47%	[34]
*Passiflora edulis Sims*	K599	GFP/RUBY	21–35	seedling without root–transgenic root	24%	[20]
*Plukenetia volubilis*	K599	RUBY/GUS/DsRed2	30	hypocotyl–transgenic root	22.7%	[18]
*Malus domestica*	K599	GFP	31	leaf–transgenic root–branch	78.8%	[39]
*Actinidia chinensis*	K599	GFP	21	leaf/hypocotyl–transgenic root–branch	66.8%	[39]
*Actinidia chinensis*	K599	GUS/eGFP	120	leaf/hypocotyl–transgenic root–branch	80%	[21]
*Ziziphus jujuba*	K599	GUS	28–42	hypocotyl–transgenic root	0.66%	[28]
*Eriobotrya japonica*	K599	GFP/RUBY	30	seedling–transgenic root	-	[35]
*Larix kaempferi*	K599	eGFP	35–40	hypocotyl–transgenic root	25.19%	[40]
*Idesia polycarpa*	K599	mCherry	35–42	hypocotyl/leaf–transgenic root–bud	71.91%	[31]
*Melia azedarach*	ATCC 15834	GUS	30–45	cotyledon, hypocotyl–transgenic root–shoot	24.0%	[29]
*Prunus dulcis*	C58C1	VENUS	60	seedling–transgenic root	75%	[41]
*Jojoba*	K599	RUBY	60	leaf blade/seedling with lateral roots and most of the leaves removed–transgenic root	27%	[42]
*Vaccinium corymbosum*	K599	EGFP	90	branch–transgenic root	74.4%	[43]
*Macadamia* spp.	K599	DsRed2	65	branch–transgenic root	28.2%	[44]

## Data Availability

The article contains all the information required to support its conclusions.

## References

[1] Xu P., Cai Y., Chen K., You R., Lu Y. (2026). *Camellia oleifera* Oil: Unveiling Health Benefits and Exploring Novel Applications. Crit. Rev. Food Sci. Nutr..

[2] Zhao Y., Xing K., Zhou J., Dong X., Qin S., Xie H., Cheng L., Yang H., Xiao J., Zhang J. (2025). Woody Oil Crop Domestication in the Genus *Camellia*: Perspectives on Future Breeding. iScience.

[3] Zhu H., Wang F., Xu Z., Wang G., Hu L., Cheng J., Ge X., Liu J., Chen W., Li Q. (2024). The Complex Hexaploid Oil-camellia Genome Traces Back Its Phylogenomic History and Multi-omics Analysis of Camellia Oil Biosynthesis. Plant Biotechnol. J..

[4] Shen T., Huang B., Xu M., Zhou P., Ni Z., Gong C., Wen Q., Cao F., Xu L.-A. (2022). The Reference Genome of *Camellia Chekiangoleosa* Provides Insights into *Camellia* Evolution and Tea Oil Biosynthesis. Hortic. Res..

[5] Gong W., Xiao S., Wang L., Liao Z., Chang Y., Mo W., Hu G., Li W., Zhao G., Zhu H. (2022). Chromosome-level Genome of *Camellia lanceoleosa* Provides a Valuable Resource for Understanding Genome Evolution and Self-incompatibility. Plant J..

[6] Lin P., Wang K., Wang Y., Hu Z., Yan C., Huang H., Ma X., Cao Y., Long W., Liu W. (2022). The Genome of Oil-Camellia and Population Genomics Analysis Provide Insights into Seed Oil Domestication. Genome Biol..

[7] Wang R., Li W., He Z., Lyu H., Wang X., Ye C., Xun C., Xiao G., Zhang Y., Zhang Z. (2025). Haplotype-Resolved Genome Assembly of the Tetraploid Youcha Tree *Camellia meiocarpa* Hu. Sci. Data.

[8] Varshney R.K., Sinha P., Singh V.K., Kumar A., Zhang Q., Bennetzen J.L. (2020). 5Gs for Crop Genetic Improvement. Curr. Opin. Plant Biol..

[9] Ma Y., Zhang Y., Zhang Z., He Z., Xun C., Wang X., Zhang Y., Wang R., Chen Y. (2025). Genome-Wide Identification and Characterization of the Xyloglucan Endotransglucosylase/Hydrolase (*XTH*) Gene Family in *Camellia oleifera* and the Function of *CoXTH1* During Drought Stress. Plants.

[10] Xiang C., Xiao L., Tao H., Cao J., Yuan J., Wang W., Jiang Y. (2025). Study on the Function of *TTG1* Gene in *Camellia oleifera*. Front. Plant Sci..

[11] Giroux B., LeBreux K., Feyzeau L., Goulet M., Goulet C., Michaud D. (2026). Gene Editing of *Nicotiana benthamiana* Architecture for Space-Efficient Production of Recombinant Proteins in Closed Environments. Plant Biotechnol. J..

[12] Wu L., Zhong L., Xiao H., Cao F., Cheng Q. (2025). Generation of Transgene-Free Homozygous Early-Flowering Poplar via Cytosine Base Editing. Plant Physiol..

[13] Tian L., Li J., Huang J. (2026). High-Efficiency Callus Induction from Intact *Camellia sinensis* Leaves via Controlled Incisions and Its Regulatory Mechanisms. BMC Plant Biol..

[14] Zhang M., Wang A., Qin M., Qin X., Yang S., Su S., Sun Y., Zhang L. (2021). Direct and Indirect Somatic Embryogenesis Induction in *Camellia oleifera* Abel. Front. Plant Sci..

[15] Lu S., Qin X., Wu M., Tao J., Guo M., Xi R., Deng X. (2026). An Efficient in Vitro Regeneration System for Adult *Camellia oleifera* Abel via Micro-Grafting. J. Appl. Res. Med. Aromat. Plants.

[16] Shen H., Chen H., Li W., He S., Liao B., Xiong W., Shen Y., Li Y., Gao Y., Li Y.Q. (2025). Development of a Robust and Efficient Virus-Induced Gene Silencing System for Reverse Genetics in Recalcitrant *Camellia drupifera* Capsules. Plant Methods.

[17] Zhang J., Tang X., Sun M., Liu C., Yu H. (2025). A Genotype-Independent Transformation and Gene-Editing System for *Populus*. Plant Biotechnol. J..

[18] Lin K., Lu L., Pan B., Chai X., Fu Q., Geng X., Mo Y., Fei Y., Xu J., Li M. (2025). *Agrobacterium rhizogenes*-Mediated Hairy Root Genetic Transformation Using Agrobacterium Gel Inoculation and RUBY Reporter Enables Efficient Gene Function Analysis in Sacha Inchi (*Plukenetia volubilis*). Int. J. Mol. Sci..

[19] Wang Y., Luo X., Su H., Guan G., Liu S., Ren M. (2024). Technology Invention and Mechanism Analysis of Rapid Rooting of *Taxus × Media* Rehder Branches Induced by *Agrobacterium rhizogenes*. Int. J. Mol. Sci..

[20] Pan J., Zheng Y., Wang T., Xiong P., Cui K., Zeng L., Fang T. (2025). Establishment of an Efficient *Agrobacterium rhizogenes*-Mediated Hairy Root Transformation System for Functional Analysis in Passion Fruit. Plants.

[21] Li P., Zhang Y., Liang J., Hu X., He Y., Miao T., Ouyang Z., Yang Z., Amin A.K., Ling C. (2024). *Agrobacterium rhizogenes*-Mediated Marker-Free Transformation and Gene Editing System Revealed That *AeCBL3* Mediates the Formation of Calcium Oxalate Crystal in Kiwifruit. Mol. Hortic..

[22] Xue C., Chang L., Liu J., Gu K., Zheng P., Wu J. (2024). Highly Efficient Adventitious Root Regeneration and *Agrobacterium rhizogenes*-mediated Hairy Root Transformation in Pear. Plant Cell Tissue Organ Cult..

[23] Ma H., Liu N., Sun X., Zhu M., Mao T., Huang S., Meng X., Li H., Wang M., Liang H. (2023). Establishment of an Efficient Transformation System and Its Application in Regulatory Mechanism Analysis of Biological Macromolecules in Tea Plants. Int. J. Biol. Macromol..

[24] Peng K., Xue C., Huang X. (2024). Enhancing Virus-Induced Gene Silencing Efficiency in Tea Plants (*Camellia sinensis* L.) and the Functional Analysis of *CsPDS*. Sci. Hortic..

[25] He Y., Zhang T., Sun H., Zhan H., Zhao Y. (2020). A Reporter for Noninvasively Monitoring Gene Expression and Plant Transformation. Hortic. Res..

[26] Prusty M.R., ShatilCohen A., Kumar R., Sharma D., MinzDub A., Ezrati S., Hihinashvili A., Sharon A. (2025). Pigments to Precision: RUBY Aiding Genetic Transformation and Genome Editing in Wheat and Barley. Physiol. Mol. Biol. Plants.

[27] Yu J., Deng S., Huang H., Mo J., Xu Z., Wang Y. (2023). Exploring the Potential Applications of the Noninvasive Reporter Gene RUBY in Plant Genetic Transformation. Forests.

[28] Yang X., Maqbool A., Zang J., Niu Y., Liu Z., Wang L., Liu M. (2025). Establishment of a Simple *Agrobacterium rhizogenes*-Mediated Hairy Root Transformation System in Sour Jujube. Sci. Hortic..

[29] Van Nguyen D., Ly L.K., Bui T.P., Nguyen T.T., Chu H.H., Do P.T. (2024). An Efficient Method for Hairy Root Transformation and Transgenic Plant Regeneration of *Melia azedarach*. Plant Cell Tissue Organ Cult..

[30] Xu S., Lai E., Zhao L., Cai Y., Ogutu C., Cherono S., Han Y., Zheng B. (2020). Development of a Fast and Efficient Root Transgenic System for Functional Genomics and Genetic Engineering in Peach. Sci. Rep..

[31] Wang H., Cheng K., Li T., Lan X., Shen L., Zhao H., Lü S. (2024). A Highly Efficient *Agrobacterium rhizogenes*-Mediated Hairy Root Transformation Method of *Idesia polycarpa* and the Generation of Transgenic Plants. Plants.

[32] Haghir-Ebrahimabadi A., Hamidoghli Y., Banaei-Asl F. (2024). The Effect of Different Strains of *Agrobacterium rhizogenes* on Hairy Root Induction in *Hypericum perforatum*. Iran. J. Rangel. For. Plant Breed. Genet. Res..

[33] Gelvin S.B. (2017). Integration of *Agrobacterium* T-DNA into the Plant Genome. Annu. Rev. Genet..

[34] Gao J., Zhang L., Huang L., Liu J. (2025). Establishment of an Efficient Genetic Transformation System and Characterization of Metabolic and Transcriptomic Landscapes of Hairy Roots in *Syringa pinnatifolia*. Sci. Hortic..

[35] Wang S., He Q., Wang L., Li J., Sun H., Song L., Huang S., Huang G., Wang S., Jing D. (2026). A Versatile Hairy Root Transformation System and Its Application in *EjLEC2* Functional Research of Loquat. Plant Cell Environ..

[36] Gutierrez-Valdes N., Häkkinen S.T., Lemasson C., Guillet M., Oksman-Caldentey K.-M., Ritala A., Cardon F. (2020). Hairy Root Cultures—A Versatile Tool with Multiple Applications. Front. Plant Sci..

[37] Yang H., Jiao Y., Sun W., Zhu T., Guo J., Zhang H. (2026). A Universal Hairy Root Transformation Protocol for Evaluating Editing Efficiency in Dicot Plants. Mod. Agric..

[38] Li J., Zhao Y., Liu J., Li C., Zhang H., Song Y., Zuo Q., Wang P., Gao K., Meng D. (2026). Rooting Conifer Genetic Research: An Accessible and Efficient Transformation System. Plant Biotechnol. J..

[39] Liu L., Qu J., Wang C., Liu M., Zhang C., Zhang X., Guo C., Wu C., Yang G., Huang J. (2024). An Efficient Genetic Transformation System Mediated by *Rhizobium rhizogenes* in Fruit Trees Based on the Transgenic Hairy Root to Shoot Conversion. Plant Biotechnol. J..

[40] Lu J., Sun N., Tian M., Zhao D., Yu Y., Jiang X., Gai Y. (2025). Fluorescent Larix Hairy Root Construction and Its Application to Gene Function in Larch. Plant Cell Tissue Organ Cult..

[41] Jedličková V., Štefková M., Sánchez López J.F., Grimplet J., Rubio Cabetas M.J., Robert H.S. (2024). Genome Editing in Almond Using Hairy Root Transformation System. Plant Cell Tissue Organ. Cult..

[42] Li B., Wang Y., Ma W., Bing J., Zhou Y., Gen Y., Gao F. (2024). Establishment of Hairy Root Transformation System for Evaluating Stress-Tolerant Gene in Jojoba. Agriculture.

[43] Wang W., Wang C., Chen J., Zheng Y., Zheng J., Gu Y., Wang Y., Liu L., Li Y., Liao F. (2026). A Simple and Efficient *Agrobacterium rhizogenes*-Mediated Root Transformation System for Highbush Blueberry. Physiol. Plant..

[44] Mo Y., Fei Y.-C., Tian X., Luo Y., Lin K., Li M., Xu J., Pang Y., Wu Y., Li K. (2026). Efficient in Planta Induction of Transgenic Hairy Roots in Macadamia Seedlings and Mature Trees Using Visual Reporters. Plants.

[45] Chen F., Wang L., Huang Q., Jiang R., Li W., Hou X., Tan Z., Lei Z., Li Q., Zeng Y. (2025). Establishment of *Agrobacterium*-Mediated Transient Transformation System in Sunflower. Plants.

[46] Kereszt A., Li D., Indrasumunar A., Nguyen C.D., Nontachaiyapoom S., Kinkema M., Gresshoff P.M. (2007). *Agrobacterium rhizogenes*-Mediated Transformation of Soybean to Study Root Biology. Nat. Protoc..

[47] Kim M.J., Baek K., Park C. (2009). Optimization of Conditions for Transient *Agrobacterium*-Mediated Gene Expression Assays in *Arabidopsis*. Plant Cell Rep..

[48] Saini R., Jaiwal P.K. (2007). *Agrobacterium tumefaciens*-Mediated Transformation of Blackgram: An Assessment of Factors Influencing the Efficiency of *uidA* Gene Transfer. Biol. Plant..

[49] Sharifova S., Prasad K.V.S.K., Cheema A., Reddy A.S.N. (2025). Genetically Encoded Betalain-Based RUBY Visual Reporters: Noninvasive Monitoring of Biological Processes. Trends Plant Sci..

